# The effects of atrazine on the microbiome of the eastern oyster: *Crassostrea virginica*

**DOI:** 10.1038/s41598-020-67851-4

**Published:** 2020-07-06

**Authors:** Adrian Britt, Megan Bernini, Benjamin McSweeney, Sony Dalapati, Sophia Duchin, Kathryn Cavanna, Nicolette Santos, Grace Donovan, Katherine O’Byrne, Sarah Noyes, Manuela Romero, Kavery Nivana Theethira Poonacha, Tara Scully

**Affiliations:** The George Washington University, Washington D.C., USA

**Keywords:** Conservation biology, Bacterial genes, Marine microbiology

## Abstract

Long-standing evidence supports the importance of maintaining healthy populations of microbiota for the survival, homeostasis, and complete development of marine mollusks. However, the long-term ecological effects of agricultural runoff on these populations remains largely unknown. Atrazine (6-Chloro-*n*-ethyl-*n*′-(1-methylethyl)-triazine-2,4-diamine), a prevalent herbicide in the United States, is often used along tributaries of the Chesapeake Bay where oyster breeding programs are concentrated. To investigate any potential effects atrazine maybe having on mollusk-prokaryote interactions, we used 16S rRNA gene amplicons to evaluate how microbial compositions shift in response to exposure of environmentally relevant concentrations of atrazine previously found within the Chesapeake Bay. The dominant bacterial genera found within all groups included those belonging to *Pseudoalteromonas*,* Burkholderia*,* Bacteroides*,* Lactobacillis*,* Acetobacter*,* Allobaculum*,* Ruminococcus,* and *Nocardia*. Our results support previously published findings of a possible core microbial community in *Crassostrea virginica*. We also report a novel finding: oysters exposed to atrazine concentrations as low as 3 µg/L saw a significant loss of a key mutualistic microbial species and a subsequent colonization of a pathogenic bacteria *Nocardia*. We conclude that exposure to atrazine in the Chesapeake Bay may be contributing to a significant shift in the microbiomes of juvenile oysters that reduces fitness and impedes natural and artificial repopulation of the oyster species within the Bay.

## Introduction

Bivalves make up 14% of the 104.3 million tons of annual global marine food production^1^. The eastern oyster, *Crassostrea virginica*, is one of the bivalve species that people in North America consume the most. Before the 1970s, wild fisheries produced the majority of the 200 thousand tons of oysters consumed annually; but with the introduction of improved farming technologies, aquaculture has increased production and now accounts for more than 75% of the oysters consumed in North America. Whether they grow naturally along their native shoreline habitats or are reared artificially, exposure to pollution threatens the health and development of oysters. Over the past century, estuaries the world over have witnessed staggering losses to oyster populations: according to one estimate, there are currently 97% fewer oysters than there were about 100 years ago^2^.

The pollutants of greatest concern include herbicides such as glyphosate and atrazine, general use pesticides such as Metam sodium, and fertilizers such as calcium nitrate. These pollutants are becoming increasingly common in aquatic systems, due to global agricultural intensification practices^3,4^. The most vulnerable areas of the Chesapeake Bay Estuary are its tributaries, which harbor the highest concentrations of pollutant contamination. Estuarine runoff from agricultural fields occurs when either farmers irrigate their fields excessively or, after a period of drought, intense rain showers saturate the fields. This excessive runoff can transport any chemical that is water-soluble into the surrounding aquatic systems. Once they reach the Chesapeake Bay estuary and its surrounding tributaries, these pollutants mix with estuarine organisms like the eastern oyster, *Crassostrea virginica*. Although the water in the Chesapeake Bay does respond to the ebb and flow of tidal movements, the elongated shape of the bay tends to trap the polluted water that reaches the estuary. Consequently, the organisms found in the bay suffer long-term exposure to pollutants such as atrazine, which is not the case with those organisms that live along coastal or other shoreline aquatic environments where the water circulates with greater force. Of the various pollutants that get trapped in the estuaries’ water, pesticides pose the greatest threat to aquatic as well as terrestrial ecosystems. Herbicides, which are a kind of pesticide, are of particular concern because they are designed to kill off what are known as producers, or the base of a food web for ecosystems.

Atrazine is the second most widely used herbicide in the world. It has been banned in the European Union, but is still used in the United States, with an estimated 76 million pounds sprayed annually on crops^5^. Atrazine (6-Chloro-*n*-ethyl-*n*′-(1-methylethyl)-triazine-2,4-diamine) is a synthetic herbicide regularly applied to crops like corn and sugar cane during the spring and summer months^6^. By design, Atrazine inhibits photosynthesis. This inhibition occurs within the plant cell chloroplast thylakoid membrane; it is triggered when atrazine binds to D1 proteins of the photosystem II complex^7^. The herbicide binding protein blocks CO2 fixation, compromising the targeted plant’s ability to produce the energy that it needs to grow^8^. The inhibition ultimately leads to the death of the susceptible plant, which generally occurs within 14–21 days after exposure. Atrazine has been known to remain in soils for at least a couple of months and even for up to several years^9^. The chemical does not break down easily in water either (half-lives > 200 days), which means that once it reaches the estuaries of the Chesapeake Bay, it can remain there for an indefinite period and in ever-increasing concentrations^6^. A study conducted by the USDA in 2006 found the highest concentration of atrazine in the Chesapeake Bay watershed to be 30 μg/L, 10×, which is at the threshold of the contaminant safety level^10^. The U.S. Safe Drinking Water Act recognizes the maximum contaminant level of atrazine to be 3 μg/L^6,10^.

### Research on Atrazine’s effects on the reproductive cycles of various species

Independent studies have shown that atrazine can cause chemical castration in frogs^11,12^. These studies indicate that male frogs that have been induced by atrazine to become females are closer to genetic females than they are to males. It has also been noted that exposure to concentrations “as low as 0.1 parts per billion” of atrazine in surface water have adversely affected frogs by causing the male gonads to produce eggs—effectually turning males into hermaphrodites^11,13^. Together, the present data suggests that sex-reversal by atrazine is an effect that occurs across nonamniote vertebrate classes^11–13^. This sex-reversal effect may be of consequence to invertebrate animals as well, particularly those that exhibit sequential hermaphroditism (changing one’s sex throughout a lifetime) such as does *C. virginica*. Further evidence of atrazine’s potential deleterious effects are evidenced in cases of human exposure, where changes in menstrual hormone levels shift follicular and ovarian phases that result in cycle irregularity^14^. Ultimately, these studies indicate a reproductive reaction to atrazine exposure.

### Research on Atrazine’s effects on infectious diseases that harm bivalves

There are very few studies examining microbiome communities in bivalves and even fewer that focus on *C. virginica.* These microbes can have positive, negative, or neutral effects on the organism. Their interactions may also change based on the composition of other microbial species, the population sizes of the other microbes, and the genetic expression of the host. The majority of oyster-prokaryote research has focused on the “protective and preventative role” of certain species of bacteria against a potentially fatal pathogen, *Vibrio*^15^. In one study, *Alteromonas haloplanktis,* isolated from scallops, “inhibited the growth of both *Vibrio anguillarum* and *Vibrio alginolyticus *in vitro”^16^. Similarly, a study concluded that *Aeromonas media* A199 protects scallops against *Vibrio tubiashii* via interactions with an aromatic heterocyclic organic compound known as indole (C8H7N)^17,18^.

According to one study, 20% of bacterial isolates from *C. virginica* are capable of producing indole (“an intracellular signal molecule which regulates various aspects of bacterial physiology, including spore formation, plasmid stability, resistance to drugs, biofilm formation, and virulence”)^19^. Another study has found that adding isolated bacteria from *C. virginica* samples to larvae that have been infected with *Vibrio corallilyticus* significantly increases the oyster’s chances of survival^20^. Finally, it has been discovered that *Phaeobacter* sp. S4, which is a bacterial isolate from the inner shell of *C. virginica*, also protects *C. virginica* larvae and juveniles challenged with *R. crassostreae* and *V. tubiashii*^21^. However much these beneficial bacterial help to defend their host, the protection they provide may be short-lived^20,21^. Additional work is required to fully comprehend the protective and possibly opportunistic role of the microbial community of *C. virginica*.

As filter-feeders, oysters are exposed to many different microbes. Since they also have unique physical structures, there are many surfaces and micro-ecosystems in which microbes can reside. It is thus unsurprising that oysters harbor an order of magnitude greater concentration of bacteria than the surrounding water in which they live^22,23^. Through next-generation microbiome analysis, we characterized the oyster’s microbial community composition and recorded a snapshot of these integral dynamics^24–28^. As we aim to demonstrate, this experiment offers a unique opportunity to improve our understanding of how oyster–prokaryotic interactions respond to ecologically relevant chemical contaminants.

In this study, we used 16s rRNA gene amplicon sequencing to characterize the microbiome of hatchery raised *C. virginica* juveniles. We sought to determine the extent to which atrazine may be affecting the bacterial composition of juvenile oysters, after having been exposed to environmentally relevant concentrations. As a herbicide commonly detected in the Chesapeake Bay Watershed^10^, atrazine was chosen to be the focus of this study investigating the effects of herbicide-induced microbiome shifts in *C. virginica* juveniles.

## Methods

### Examined concentrations

This study used a range of atrazine concentrations based upon those commonly detected in the Chesapeake Bay^10,29–33^. The following outlines the chosen concentrations and the reasoning for using that concentration in the experiment:

1. 30 µg/L Atrazine: The peak concentration of atrazine detected in any sample within the Chesapeake Bay was 30 µg/L (station L0002488), which is located in a tributary on the Eastern Shore^10^. The highest concentration recorded is of interest, as it represents an extreme concentration.
2. 20 µg/L Atrazine: A half-way point in between 30 µg/L Atrazine and 10 µg/L Atrazine for data exploration purposes.
3. 10 µg/L Atrazine: During June of 2011, atrazine concentrations from the upper Choptank river were logged. The average of all recorded concentrations was 0.29 µg/L. The highest recorded concentration was 10 µg/L^10,13,29^. The general trend in the data indicates that the main detections of atrazine are in the tributaries while significantly lower concentrations have been found in the Bay itself. Oyster restoration grounds are generally found within tributaries along the upper, middle and lower sections of the Choptank river^30^.
4. 3 µg/L Atrazine: The EPA’s Maximum Residue Limit^10,13^ for atrazine is relevant because it is the limit which the EPA has set forth for regulating our own drinking water, further investigation into the MRL of atrazine is merited based on health concerns cited in many scholarly papers throughout the last decade, the last official measurement was carried out in 2017^30–32, 34–37^.
5. 30 µg/L Acetone: Atrazine stock solution was dissolved in a 100% acetone solution. In order to assume continuity throughout the experiment this treatment was added to assure that any noticed effect on development was due solely to atrazine exposure.
6. 0 µg/L Atrazine/Acetone: Control group.

### Oyster acquisition and stabilization

300 juvenile *Crassostrea virginica *oysters of similar size and weight were purchased from Horn-Point Laboratory. The oysters were separated into six groups of 50 specimens and randomly assigned to an exposure group (30 µg/L Atrazine, 20 µg/L Atrazine, 10 µg/L Atrazine, 3 µg/L Atrazine, 30 µg/L Acetone and a atrazine and acetone-free Control of instant ocean seawater). 3.0 mm square mesh sieves were used to separate each group. No oyster was smaller than 5.0 mm long × 4.0 mm wide when placed in the mesh sieves. Oysters underwent a 2-week long acclimation period inside of a large holding tank filled with 300 L of pressure filtered instant ocean seawater at a salinity of 25 parts per thousand (ppt). Frequent water changes (25% twice weekly) were used in order to minimize buildup of both ammonium and nitrate within the closed water system. Each oyster group was fed 6 L of a concentrated phytoplankton mixture of (*Tetraselmis Chuii*,* isochrysis galbana*, and* Nannochloropsis oculata*) approximately ~ 400,000 cells/mL) every day.

Relevant tank water parameters were monitored every other day and water changes were completed when required. The tank was consistently maintained to fit the following water parameters:

**Table:** 

Salinity (ppt)	Ammonium (ppb)	Nitrate (ppb)	Plankton concentration
25	0	0	> 9,000 cells/ml

Water quality parameters were monitored using: Salinity Refractometer—Salinity, API Testing Kit—Ammonium levels, API Testing Kit—Nitrate levels, Mass Spectrophotometer at 654 nm—Plankton Concentration, Hemocytometer count—Plankton Concentration.

### Assigning experimental groups

Individual oysters were randomly separated into six groups containing 50 oysters each. To prevent potential overcrowding of the organisms and ensure that each oyster in the study was filtering the same amount of water, each of these groups was subsequently divided into a sub-group of 10 oysters and placed into 3.0 mm square mesh sieves. These sub-groups of 10 oysters were kept together throughout the entirety of the experiment. The acetone group was used because the atrazine we used for the experiment was diluted in a solution of HPLC-grade acetone. Each group of 50 oysters was assigned to one of six treatments: 0 μg/L Atrazine/Acetone, 3 μg/L Atrazine, 10 μg/L Atrazine, 20 μg/L Atrazine, 30 μg/L Atrazine, and 30 μg/L Acetone.

### Exposures

Following the 2-week long stabilization period, the experimental groups were exposed to their first atrazine trial. All oyster groups were kept in separate tanks to avoid any potential cross contamination. In order to expose the oyster groups to atrazine and acetone, each group was removed from its holding tank and placed into a separate 4-L glass tank containing 2 L of newly made seawater (salinty = 25 ppt). Atrazine was added to each 4-L glass tank according to the corresponding treatment concentration. In order to mimic the heavy rainfall pattern surrounding the Chesapeake Bay^38^, treated groups spent a total of 3 h submerged in the treated 2 L of saltwater three times per week, every other day (Monday, Wednesday, Friday). Aeration was provided in both holding and treatment tanks.

In order to minimize any potential contamination of each group’s respective holding tanks after the 3-h exposure period, each group was washed using a constant stream of pressure-filtered water for three one-minute rinses. Oyster groups were then relocated to their respective 40-L glass bio-cube tanks. The bio-cube tanks were set to have the same parameters as the large holding tank.

### Prokaryotic DNA extraction

Each oyster was carefully opened using sterilized forceps. Whole tissue extraction was done by scraping and removing all existing tissue from each of the two shells. The whole oyster tissue was then placed in a labeled 1.5 mL microcentrifuge tube with the treatment and group to which it belonged. In order to extract only the prokaryotic DNA a QIAGEN QIAamp DNA Microbiome Kit protocol was used.

### Oyster weights

Oysters were weighed on a weekly basis using a Mettler Toledo PB303-S Digital Balance Scale MonoBloc Weighing Technology. During each weighing session, every oyster group within each of the six treatments was carefully removed from the holding bags and placed into a dish (previously weighed separately in order to set scale to zero). After placing the dish with the oysters on the scale, oyster weights were recorded to the nearest ten thousandth place. Oysters were then removed from the dish and laid out on a surface for pictures. All previous steps were repeated for each of the groups within the six treatments. Pictures of each group were taken independently to make sure that the correct number of specimens were there. All weights were recorded in grams. The last set of weights (g) were recorded on September 25, 2018, the same date on which five out of the ten oysters belonging to each of the six treatment groups were removed for DNA isolation. Following the removal of half of the oysters in each treatment group, the remaining five specimens in each group were kept under the same environmental conditions, except that they were no longer exposed to atrazine. All the dates under the post-atrazine treatment section of the graph therefore belong only to the remaining five oysters from each group; in order to account for this loss, group averages were taken and divided by the total number of individuals per group. September 30, 2018 marks the last day on which oyster weights (g) were recorded.

### 16S sequencing

The following protocol was produced and completed with CD Genomics.

Amplicons were sequenced on a paired-end Illumina HiSeq2500 platform to generate 250 bp paired-end raw reads, and then pretreated. The forward and reverse primers used were 338F:5′ ACTCCTACGGGAGGCAGCA-3′ and 806R:5′- GGACTACHVGGGTWTCTAAT-3′ respectively. Specific processing steps for both sequencing events were completed as follows: (1) Paired-end reads were assigned to a sample by their unique barcode, and the barcode and primer sequence were then truncated. (2) Paired-end reads were merged using FLASH (V1.2.7, https://ccb.jhu.edu/software/FLASH/), a very fast and accurate analysis tool to merge pairs of reads when the original DNA fragments are shorter than twice of the reads length. (3) Quality filtering was then performed on the raw tags under specific filtering conditions of Trimmomatic v0.33 (https://www.usadellab.org/cms/?page=trimmomatic) quality control process. After filtering, high-quality clean tags were obtained. (4) The resulting high quality tags were compared with the reference database (Gold database, https://drive5.com/uchime/uchime_download.html) using UCHIME algorithm (UCHIME Algorithm (https://www.drive5.com/usearch/manual/uchime_algo.html) to detect chimeric sequences and then the chimeric sequences were removed.

### Statistical analysis

The following protocol was produced and completed with CD Genomics.

To characterize microbial communities and to perform functional analyses, the following wrappers were employed in R statistical software: summarize_taxa_through_plots (to produce the taxonomical files and charts), alpha_rarefaction and beta_diversity_through_plots (to assess respectively the alpha- and beta-rarefaction diversity indices), and principal_coordinates.py (to compare groups of samples based on phylogenetic distance metrics). To compare treatment groups (Control versus all treatment concentrations), AMOVA (analysis of molecular variance) analyses was performed at genus level (P-value < 0.005; FDR < 0.01) using Excel version 16.13. In order to further test for significant variation between groups in the metagenomic data, METASTATS (a software for detecting differentially abundant features in metagenomic case/control studies) was used. It is the first tool developed in the context of metagenomic data comprising multiple samples and relies on a non-parametric t-test^39^. Finally, a heatmap analysis was also created via clustering of high abundance and low abundance genera. The heatmap was aggregated by color gradient and similarity to reflect the similarity and difference of the bacterial composition of multiple samples. “According to the taxonomic composition and relative abundance of each sample, the genera heatmap analysis was carried out to extract the species at each taxonomic level and plotted using the R language tools, the heatmap clustering analysis was conducted at the level of phylum, class, order, family, genus and species respectively” (CD Genomics).

## Results

### Diversity of microbial communities and taxonomic richness

**Figure 1:**
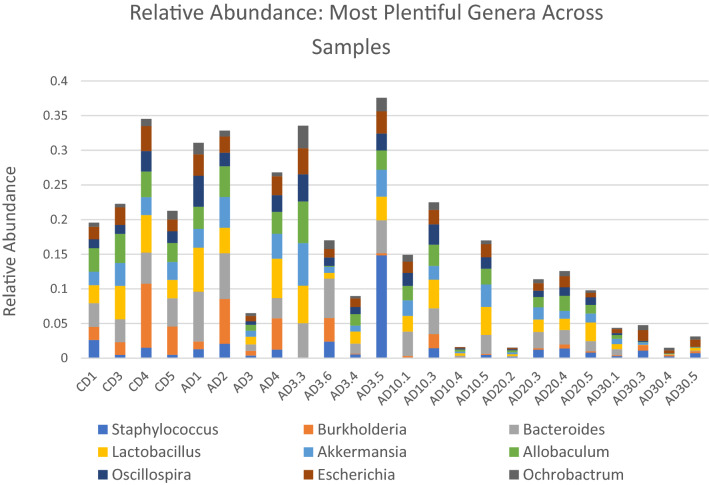
Abundance of bacterial genera observed in each sample and across groups. The groups are depicted from left to right as follows: CD 1–5: Control; AD 1–4: Acetone; AD 3.3–3.6: Atrazine 3 µg/L; AD10.1–AD10.5: Atrazine 10 µg/L; AD20.2–AD20.5: Atrazine 20 µg/L; AD30.1–30.5: Atrazine 30 µg/L.

As seen in Fig. 1, the relative abundance of the most plentiful genera found between all samples was significantly decreased in specimens exposed to concentrations of atrazine higher than 10 µg/L. According to the AMOVA test scores, this decrease was most significant in the Control vs 30 µg/L (p < 0.001). The same overall decrease in abundance holds true for bacterial composition on the species and phyla levels (supplementary material S1, S3). All groups included dominant bacteria from the orders *Rickettsiales*, *Vibrionales*, and *Alteromonadales*.

**Figure 2:**
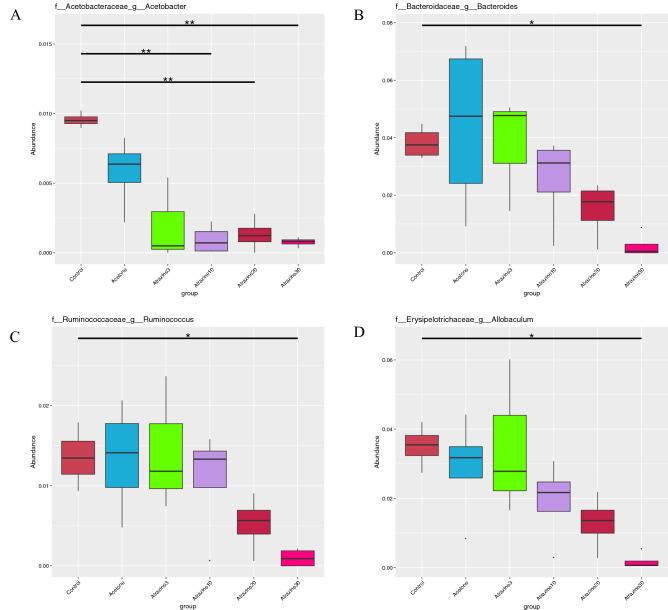
The box graph visually displays the results of the METASTATs, t-tests with sample permutation for detecting differentially abundant features in a metagenome. A non-parametric T-test determines whether there are any taxa that are differentially represented between samples; significance is depicted by the asterisks. Plots were made in R, the first name listed refers to the bacterial family, the second the genera. The vertical axis denotes relative abundance.

As seen in Fig. 2A,B, both *Acetobacter* and *Bacteroides* abundance dropped to negligible levels if the animal was treated with 30 µg/L of atrazine. In Fig. 2C,D we find similar reductions in abundance within Ruminococcus and Allobaculum, respectively. *Allobaculum*, *Oscillospira*, *Escherichia*, and *Bacteroides* predominate amongst the control samples. We detected a significant dose-dependent relationship to relative abundance for both *Bacteroides* and *Allobaculum*: as oysters were exposed to higher concentrations of atrazine, a decrease in their relative abundance was observed*.* Acetobacter and Ruminococcus experienced a particular loss of relative abundance when exposed to concentrations of atrazine higher than 10 µg/L.

**Figure 3:**
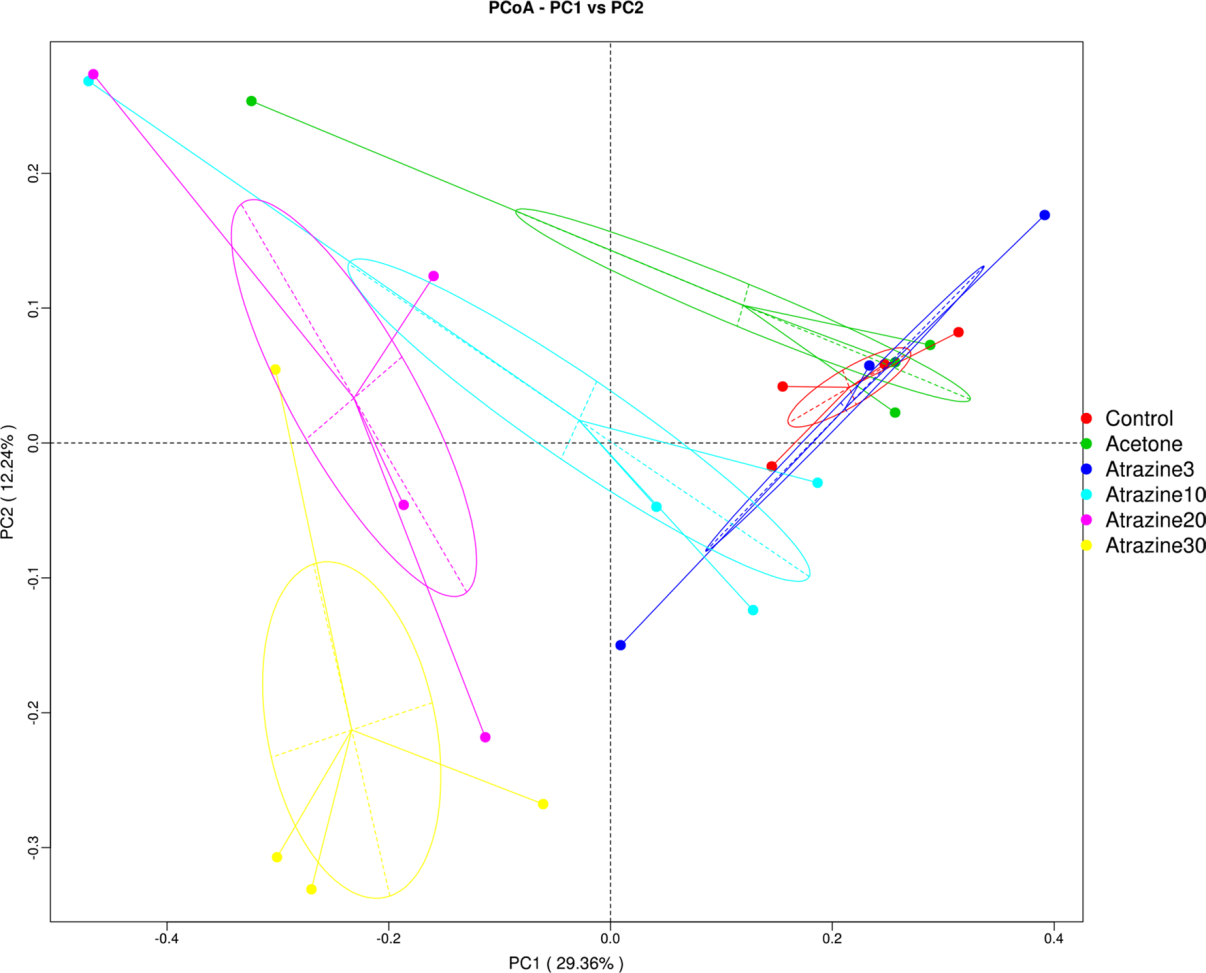
PCoA plot: each point represents a sample, plotted by a principal component on the X-axis and another principal component on the Y-axis, which was colored by group. The percentages on each axis indicate the contribution value to discrepancy among samples.

A principal coordinate analysis (PCoA) was performed using unweighted UniFrac distances (Fig. 3). We used these analyses to test for significant differences in microbial genera composition between all groups. Figure 3 shows a clear grouping along both the x-axis (PC1 = 29.36% of variability) and the y-axis (PC2 = 12.24%) between the control, acetone and 3 µg/L atrazine samples. The remaining treatment groups (atrazine 10 µg/L, 20 µg/L and 30 µg/L, respectively) clustered farther away with increasing dosages of atrazine.

**Figure 4:**
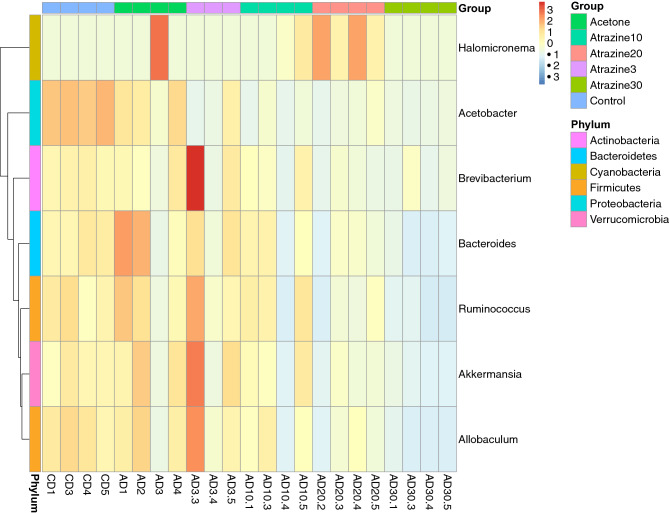
In the heatmap analysis the vertical clustering indicates the similarity of the abundance between different genera. The shorter the distance between the two genera, the more similar abundance between the samples. In the horizontal clustering, the closer and shorter of the branch length between the samples, the more similarity of the abundance.

Similarly, the acetone and control groups were found to contain more similar microbial compositions than any of the atrazine groups did (Fig. 3). However, 30 µg/L atrazine is the only group found in the bottom left quadrant of the plot, indicating this treatment results in the most dissimilar microbial composition. According to the heatmap in Fig. 4, *Allobaculum*, *Akkermansia*, *Ruminococcus,* and *Bacterioides* all severely decreased in the atrazine 30 µg/L group. However, *Allobaculum*,* Akkermansia*,* Ruminococcus*,* Bacteroides*,* Brevibacterium*,* Aceobacter*, and* Halomicronema genera *all showed decreased levels of abundance in the oyster groups treated with atrazine (Fig. 4).

An analysis of molecular varience (AMOVA) test was performed on all treatment groups for the most abundant genera (Table 1).

**Table 1:** AMOVA results for weighted unifrac distances.

VS Group	SS	df	MS	Fs	p-value
Atrazine20-Control	0.40281 (0.355577)	1 (6)	0.40281 (0.0592628)	6.79701	0.001*
Atrazine30-Control	0.460711 (0.533829)	1 (6)	0.460711 (0.0889715)	5.17818	< 0.001*
Atrazine20-Atrazine3	0.375038 (0.415643)	1 (5)	0.375038 (0.0831285)	4.51154	< 0.001*

Asterisk marks significance in microbial communities between groups.

*Psuedoalternamonas*,* Fusobacterium*,* Bacterioides* and *Clostridium* were found to be present within both Atrazine 20 and Atrazine 30 groups, but at significantly lower levels when compared to the control. Additionally, a significant difference was detected between the Atrazine 20 group when compared to Atrazine 3 group when comparing between the most abundant genera.

Finally, bacteria in the genus *Nocardia* were detected only in groups exposed to atrazine (S2). These differences in microbial composition may shed some light on the differences between each groups’ average growth over the experiment.

### Oyster growth data

**Figure 5:**
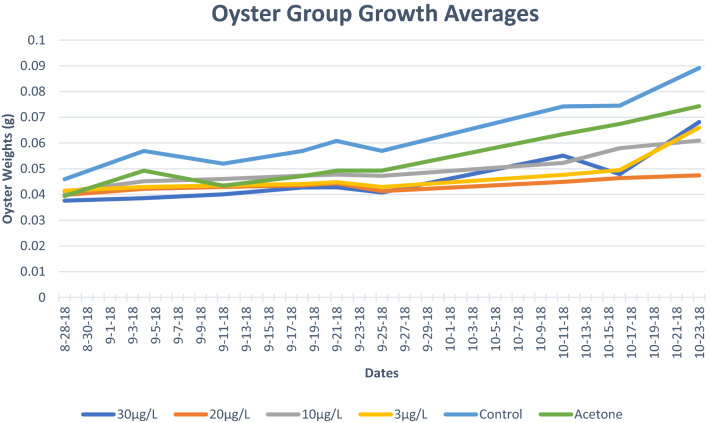
Line graph depicting the average growth in grams of the different oyster groups. The red line dividing the graph symbolizes the end of the atrazine treatment.

The general trend when comparing slope values was that as atrazine concentrations increased, slope values tended to decrease (Fig. 5). Both the control (0.0371) and acetone (0.0318) treatments displayed the highest slope values (Fig. 5). The highest slope values correlate to the treatment groups with the highest average weights over time, which in this case is approximately over the course of 3 months. The Atrazine group with 10 µg/L experienced the fastest growth when compared to the other atrazine treatment groups. The atrazine 30 µg/L group was expected to have undergone the least growth as it was the group that was exposed to the most elevated concentration; however, the Atrazine 20 µg/L treatment group had the lowest slope value (0.0128). On October 16, 2018, the Atrazine 30 µg/L average growth line drops, which could be attributed to the death of an oyster that was not detected at the time (Fig. 5).

## Discussion

### Interpreting the results

The present study investigates the effects of atrazine exposure on the microbiome of *C. virginica*. Today, we understand that the microbiome of marine mollusks is vitally important to their survival, homeostasis, and overall development^40^. The microbiome of filter-feeding bivalves such as oysters is comprised of both continuously occupant and temporary transitory microbiota. Eastern oysters are suspension-feeders and “filter large quantities of water (e.g., 3–9 L/h/g dry mass for oysters”^41,42^. As such, they are exposed to both free-living and particle-associated microbes. Therefore, the oysters’ microbiome may be particularly important for physiological and biochemical enantiostasis (open system maintenance).

Our results indicate that oysters can act as a host for a specific set of core microbiota. Although the oysters in the study were comprised of hatchery-reared juveniles, many of the bacteria sequenced in this study were also found residing within oysters raised and sampled in the wild^43–46^. Our results also indicate that atrazine exposure may cause *C. virginica* juveniles to develop at a slower rate than they would without exposure, possibly due to a loss of beneficial bacteria (Fig. 5).

The microbial composition of the control group’s four samples bear a greater resemblance among themselves than when they are compared to those other groups that had been exposed atrazine (Fig. 3). This may be a sign of a healthy core microbiome existing within the control groups but not in the atrazine exposed experimental groups. It may be that as the core microbiome’s relative abundance decreases, it gives way to new, potentially pathogenic bacteria which take hold within the animal. According to the PCOA, this was particularly evident in those groups that were exposed to at least 3 μg/L.

A significant increase of *Nocardia* (a gram-positive, partially acid-fast actinomycete, pathogenic bacteria) was observed in the 3, 10, 20, and 30 μg/L atrazine treatment groups. A subsequent decrease in *Nocardia* was detected in the 30 μg/L treatment group, which may account for this group’s increased growth rate between 10/16/18 and 10/23/18. Nonetheless, *Nocardia* was found only within the atrazine treated groups (Supplementary material S2), which suggests the presence of atrazine may have artificially selected for the colonization and subsequent survival of pathogenic *Nocardia spp*.

*Nocardia* is often found in aquatic and terrestrial environments, existing as soil saprophytes, but can also appear in fresh and saltwater, dust, decaying vegetation and fecal deposits from animals. *Nocardia* was found to be lethal to *Ostrea edulis,* flat oysters^45–48^ and may also be lethal to *C. virginica* in advanced stages of colonization^49^. In a study by Carella et al., *Nocardia crassostreae* was observed in *Mytilus galloprovincialis* and *Ostrea edulis* sampled from raft-culture farms in the Gulf of Naples^49^. Carella et al. found that the inflammatory lesions observed were characteristics also found in other *Nocardia*-related cases in different animals, both in invertebrates and vertebrates alike. However, there have been no other studies that compare and confirm these findings.

The microbial communities of oysters include both resident (or core) as well as transient microbiota. Oysters are thus highly susceptible to pathogen introduction and atrazine may be affecting the ability of beneficial oyster microbes to fight off both known and unknown bacterial pathogens. One of the beneficial roles of the oyster microbiome is to defend the host oyster against pathogen inoculation and environmental stress^43^. However, in compromised hosts, the helpful microbes may well become opportunistic pathogens^50–52^.

*Psuedoalternamonas*, *Fusobacterium* and *Clostridium* species, which all form part of *Crassostrea virginica*’s core microbiome, were found to a lesser degree within the 20 μg/L atrazine treated sample when compared to the control (Table 1). This finding indicates that atrazine exposure may play a role in crafting an environment which does not permit *Psuedoalternamonas*, *Clostridium* and/or *Fusobacterium *species to effectively colonize the oysters during their juvenile life stage, a stage of rapid organismal development. There have been little to no studies done investigating the effects these bacteria can have on oysters, which means that the reduction of these bacteria could have a positive, negative, or neutral effect on the oysters. However, these groups have often been found to be part of the microbiome of bivalves worldwide^53–55^.

### Known bacterial interactions

Studies have found that *Proteobacteria *and *Cyanobacteria* are integral members of bivalve microbiomes, irrespective of their host^56,57^. *Bacteroides* species normally form mutualistic relationships within the gut of bivalve species, where they process complex molecules into simpler ones within the host intestine, and at times by produce indole^58–60^. Our results indicate that atrazine has decoupled this interaction by decreasing *Bacteroides* relative abundance in particular (Figs. 1, 4). It may be possible that *Bacteroides* indole production helps to keep pathogenic bacteria from colonizing the oyster microbiome. However, these genera are not the most plentiful within the microbiome of oysters. In multiple studies, and in particular one conducted by Wexler, H. M, it was found that *Proteobacteria* can account for a majority of the total abundance^33,57,61–64^. Other dominant phyla that have been identified include *Acteroidetes*, *Actinobacteria*, *Firmicutes*, *Chlamydiae*, *Fusobacteria, Spirochates, Chroloflexi, Plantomycetes,* and *Verrucomicrobia*^12,48,56, 65,66^. Our study corroborates these findings (S3).

*Achromobacter*, *Aeromonas*, *Flavobacterium*, *Micrococcus*, and *Vibrio* are all bacterial genera commonly isolated from bivalves^53,60, 67–70^, however, more recent culture-independent work has shown that while these genera are commonly present, they do not necessarily represent most of the community^54,55,71^. We detected all of the above genera within our samples except *Aeromonas*. Because these genera do not represent the majority of the community, it is unsurprising that we did not detect all of the bacterial genera known to occur in oyster microbiomes.

## Conclusion

Because the microbiome of *C. virginica* is vital for its homeostasis and survival—especially during its developmental stages—the persistence of atrazine in the Bay may increase the fatality levels of juvenile oysters by selecting for the colonization of pathogenic bacteria and the subsequent decrease in the relative abundance of common beneficial microbes. This interaction may also be affecting the growth rate of *C. virginica* juveniles, causing them to grow at a slower rate. If so, atrazine contamination can significantly harm the Chesapeake Bay’s oyster restoration efforts, which target the reseeding of baby oysters and not adults. More research must be done on the long-term effects of this herbicide, but our findings indicate that growing atrazine concentrations in the Chesapeake Bay may be accelerating the drastic decline of this keystone species. This study’s sequence data could be used to compare oyster populations along the eastern seaboard as well as the Gulf Coast where the eastern oyster grows naturally and is reared in aquaculture farms. These microbiome shifts may not be isolated to just herbicide exposures; thus, further research into individual and combined effects of other contaminants would pinpoint which ones are the most detrimental to healthy microbial communities.

This research should expand to sample more time points, repeat herbicidal challenges with the inclusion of pathogens, and sequence molecular data to determine the oyster’s response to both challenge treatments and shifts in microbial community composition. This is a vital baseline for future research aimed at understanding the role of the microbiome in oyster development and physiology. As such, future research efforts should also focus on more robust sampling in natural habitats, working with oyster farmers, hatcheries, and restoration labs to help revitalize the oyster population.

## Supplementary information

Supplementary file1
