# Supplementary material for: The effects of atrazine on the microbiome of the eastern oyster: Crassostrea virginica
Source: Sci Rep. 2020 Jul 6;10:11088. doi: 10.1038/s41598-020-67851-4 (PMC7338443; doi:10.1038/s41598-020-67851-4)

Authors:
Adrian Britt, Megan Bernini, Benjamin McSweeney, Sony Dalapati, Sophia Duchin, Kathryn Cavanna, Nicolette Santos, Grace Donovan, Katherine O'Byrne, Sarah Noyes, Kavery Nivana Theethira Poonacha, Manuela Romero, Dr. Tara Scully

The Effects of Atrazine on the Microbiome of the Eastern Oyster: *Crassostrea virginica*

Supplementary Material

S1: Relative abundance of most prevalent bacterial species across all groups:

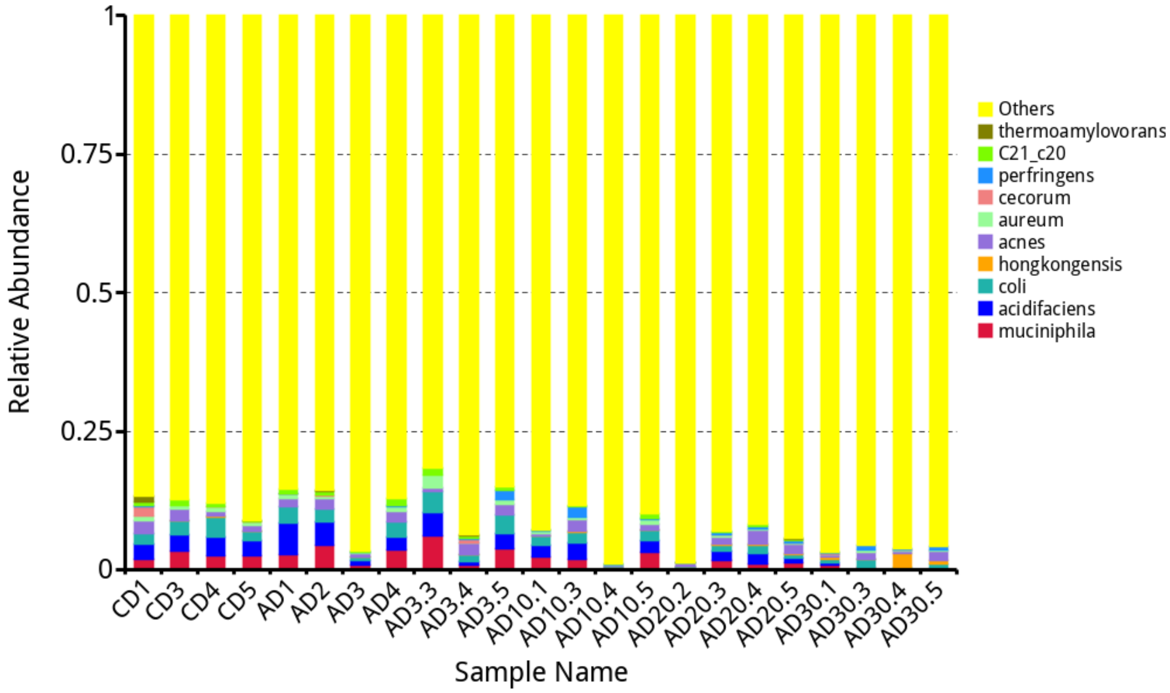

S2: Relative abundance of *Nocardia* across all groups:

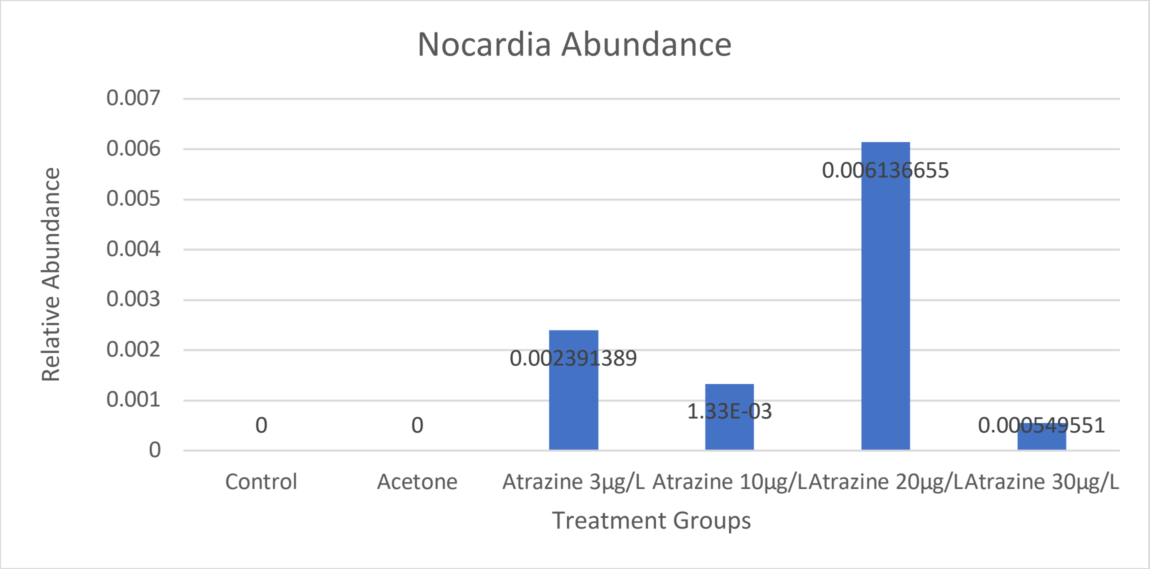

S3: Relative abundance of most prevalent bacterial phyla across all samples:

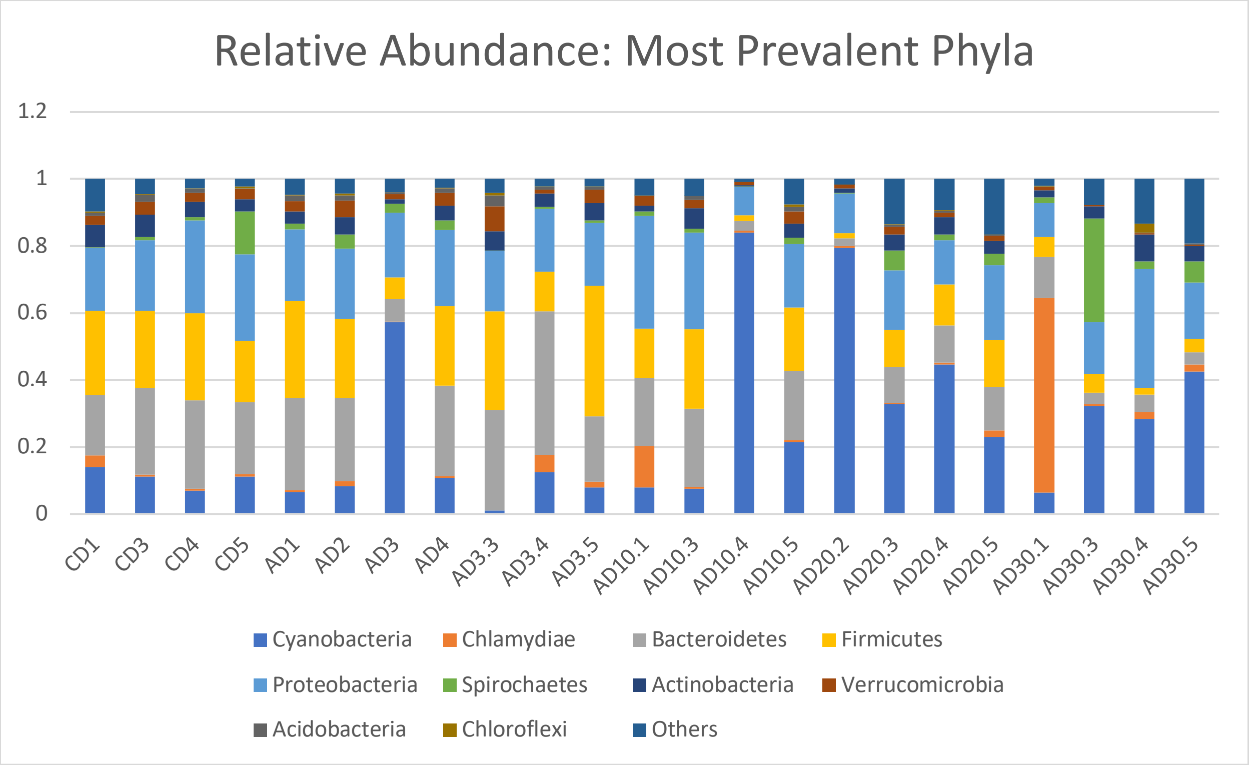

Supplement: Supplementary file 1 — Supplementary file1 [file 41598_2020_67851_MOESM1_ESM.pdf]
